# *In vitro* Fermentation Reveals Changes in Butyrate Production Dependent on Resistant Starch Source and Microbiome Composition

**DOI:** 10.3389/fmicb.2021.640253

**Published:** 2021-04-29

**Authors:** June Teichmann, Darrell W. Cockburn

**Affiliations:** Department of Food Science, The Pennsylvania State University, University Park, PA, United States

**Keywords:** resistant starch, gut microbiome, fermentation, butyrate, 16S, pH, personalized diet

## Abstract

One of the primary benefits associated with dietary resistant starch (RS) is the production of butyrate by the gut microbiome during fermentation of this fiber in the large intestine. The ability to degrade RS is a relatively rare trait among microbes in the gut, seemingly confined to only a few species, none of which are butyrate producing organisms. Thus, production of butyrate during RS fermentation requires a network of interactions between RS degraders and butyrate producers. This is further complicated by the fact that there are multiple types of RS that differ in their structural properties and impacts on the microbiome. Human dietary intervention trials with RS have shown increases in fecal butyrate levels at the population level but with individual to individual differences. This suggests that interindividual differences in microbiome composition dictate butyrate response, but the factors driving this are still unknown. Furthermore, it is unknown whether a lack of increase in butyrate production upon supplementation with one RS is indicative of a lack of butyrate production with any RS. To shed some light on these issues we have undertaken an *in vitro* fermentation approach in an attempt to mimic RS fermentation in the colon. Fecal samples from 10 individuals were used as the inoculum for fermentation with 10 different starch sources. Butyrate production was heterogeneous across both fecal inocula and starch source, suggesting that a given microbiome is best suited to produce butyrate only from a subset of RS sources that differs between individuals. Interestingly, neither the total amount of RS degraders nor butyrate producers seemed to be limiting for any individual, rather the membership of these sub-populations was more important. While none of the RS degrading organisms were correlated with butyrate levels, *Ruminococcus bromii* was strongly positively correlated with many of the most important butyrate producers in the gut, though total butyrate production was strongly influenced by factors such as pH and lactate levels. Together these results suggest that the membership of the RS degrader and butyrate producer communities rather than their abundances determine the RS sources that will increase butyrate levels for a given microbiome.

## Introduction

Increasingly it is being recognized that a healthy gut microbiome is important to overall human health. While it is still an active area of research as to what constitutes a healthy microbiome, a high capacity to ferment dietary fiber to short chain fatty acids is one aspect that is nearly universally agreed upon. Resistant starch (RS) is starch that is able to resist digestion by human enzymes and thereby survive transit to the colon where it can be fermented by the microbial population that resides there (DeMartino and Cockburn, 2019). Consumption of RS has been linked to improved glucose control (Maziarz et al., 2017; Sandberg et al., 2017; Stewart and Zimmer, 2017), decreased serum cholesterol (Yuan et al., 2018), improved cardiovascular health markers (Peterson et al., 2018) and improvement of biomarkers in chronic kidney disease (Esgalhado et al., 2018; Khosroshahi et al., 2019). While some of the benefits of adding RS to a diet are independent of its fermentation in the gut (Bindels et al., 2017), others have been tied to its modulation of the gut microbiome (Laffin et al., 2019; Snelson et al., 2019). RS has long been noted as a standout among fibers for its ability to induce the production of butyrate (Smith et al., 1998), the short chain fatty acid (SCFA) with the strongest connection to health benefits. Butyrate is an important anti-inflammatory signal in the gut (Bach Knudsen et al., 2018) and is connected to improved gut barrier function (Yan and Ajuwon, 2017; Zheng et al., 2017), suppression of colon cancer (Li et al., 2018) and is the preferred energy source of colonocytes (Roediger, 1980).

Interestingly the number of different bacterial taxa that reside in the human colon and are able to efficiently degrade RS has been found to be fairly limited. Certain species of *Bifidobacterium*, most prominently *B. adolescentis*, and the Firmicutes *Ruminococcus bromii* are the only species that have been conclusively demonstrated to extensively degrade RS (Ze et al., 2012; Duranti et al., 2014). Some other species like *Eubacterium rectale* have some minor ability to grown on RS sources (Cockburn et al., 2015, 2018), but have their growth greatly enhanced when co-cultured with a true RS degrader (Ze et al., 2012). Both *B. adolescentis* and *R. bromii* have extensive complements of starch degrading enzymes and in the case of *R. bromii* these have been found to organize into a multi-enzyme complex termed the amylosome (Ze et al., 2015; Mukhopadhya et al., 2018), analogous to the cellulosomes utilized by other anaerobic bacteria for plant cell wall deconstruction. However, in neither case are these RS degraders butyrate producing organisms. *B. adolescentis* produces a mixture of acetate, ethanol and lactate (Amaretti et al., 2007) while *R. bromii* produces a mixture of acetate, ethanol and formate (Crost et al., 2018), with the ratios in each case dependent on carbohydrate source, stage of growth and fermentation conditions. Thus, the production of butyrate during RS fermentation is dependent on a network of cross-feeding interactions between RS degraders and butyrate producers, most prominently *Faecalibacterium prausnitzii*, a member of the Ruminococcaceae, as well as members of the genus *Roseburia* and *E. rectale* within the Lachnospiraceae. Note that it has recently been proposed to move *E. rectale* to the genus *Agathobacter* as it is not truly a member of the genus *Eubacterium* and is quite closely related to the *Roseburia* genus (Rosero et al., 2016). These bacteria are generally adept at fermenting a range of mono- di- and oligosaccharides to butyrate, often with some co-consumption of acetate, utilizing the acetyl-CoA pathway of butyrate production via the butyryl-CoA:acetate CoA-transferase (Duncan et al., 2002). Other bacteria, such as *Anaerostipes hadrus* and *Anaerobutyricum hallii* (Duncan et al., 2004) within the Lachnospiraceae are able to consume large amounts of lactate and acetate along with some carbohydrate to produce butyrate via a similar pathway. Thus, a combination of acetate, lactate and oligosaccharides released by the RS degraders can be converted to butyrate by the gut butyrate producing organisms.

Despite the relative ubiquity of RS degraders and butyrate producers within human gut microbiomes, consumption of RS does not universally lead to an increase in butyrate (McOrist et al., 2011; Venkataraman et al., 2016). The reasons for this are currently unclear and there are numerous possible reasons. There could be a lack of RS degraders, a lack of butyrate producers (or specifically lactate to butyrate producers), competition for cross-feeding products from non-butyrate producers, conditions that shift butyrate producers to other fermentation modes (most can produce things other than butyrate), influences of other dietary components or efficient absorption of the produced butyrate before the feces exits the body. A further complication is that there are many different sources and forms of RS, only united in the fact that human enzymes cannot efficiently degrade them (DeMartino and Cockburn, 2019). Type 1 RS (RS1) is starch that is surrounded by other material, such as in whole grains, rendering it inaccessible to the human enzymes. Type 2 RS (RS2) is starch found in the B-type or C-type crystalline form, most often found in tubers and legumes, respectively. Certain varieties of maize make high amylose (HAM) starch variations that also take on the B-type crystalline structure and are resistant to digestion. Note that the potato and legume starches are not high amylose, they take on the B- and C-type crystalline structure for other structural reasons. Type 3 RS (RS3) is retrograded starch, which has been cooked to lose its original structure, but re-crystallizes into a more resistant form upon extended cooling. This is the process underlying the staling of many cooked starchy products and can occur whether the parent starch was a RS or not. Type 4 RS (RS4) is chemically modified starch that has been rendered resistant, often through cross-linking reactions. All of these RS types have been found to have somewhat differential impacts on the gut microbiome and indeed two different starches within the same type can also have differential impacts (Baxter et al., 2019b; Deehan et al., 2020). Therefore, in an effort to try and disentangle some of these complexities underlying butyrate responses to RS supplementation we have undertaken an *in vitro* fermentation approach. Taking a set of 11 different fecal inocula (from 10 individuals) we have performed fermentations with each on a panel of 10 different starches, eight of which are RS sources, spanning RS2, RS3, and RS4. Changes in pH, organic acid production and composition of the microbiome were measured in comparison to a starch-free control. We hypothesized that microbiomes from different individuals would exhibit unique profiles of resistant starches that increase butyrate production. Indeed, the results show stark individuality in the responses to each starch, but with some links to key taxa within the microbiome.

## Materials and Methods

### Starches Used in This Study

For a descriptions and RS type of each starch used in this study see Supplementary Table 1. Non-resistant starches used in this study were potato amylopectin (Ap) and corn starch (CS), both from Sigma-Aldrich (St. Louis, MO, United States). For the resistant starch sources, green banana flour (Bn) was from Blue Lily Organics (Phoenix, AZ, United States), Hi-Maize^®^ 260 resistant starch (HAM2) and VERSAFIBE^TM^ 2470 resistant starch (HAM4) were from Ingredion Incorporated (Westchester, IL, United States), potato starch was from Bob’s Redmill (Milwaukie, OR, United States), ActiStar RT tapioca resistant starch was from Cargill (Wayzata, MN, United States) and the tiger nut flour (Tn) was from Organic Gemini (Brooklyn, NY, United States). Retrograded potato starch was produced according to a previously published protocol (Sharma et al., 2016). Russet potatoes were washed and peeled. Half were used for retrograded starch production with whole foods (RS3_Wh) and half were used for retrograded starch production with extracted starch (RS3_Ex). For RS3_Wh, potatoes were cut into ∼1.5” cubes and boiled for 15 min, then mashed with a mortar and pestle until no clumps remained. RS formation was induced by cooling in the refrigerator (4°C) and reheating in a 110 W microwave oven twice for 1 min at 100% power (power level 10) (model number JES1351WB, GE, Boston, MA, United States). For RS3_Ex, starch was first extracted from the potatoes, following a previously published protocol (Krunic et al., 2018). Briefly, the potatoes were ground up in a food processor (Mini-prep PLUS, Cuisinart, Stamford, CT, United States), reduced in 4 mM sodium sulfate and rinsed using 10 volumes of 50 mM sodium phosphate buffer (pH 6.5) and deionized water. After centrifuging, the starch (sediment) was left to dry at room temperature. The RS3_Ex was then treated to the same boiling, cooling, and reheating scheme as the RS3_Wh samples. The RS content of these starches was determined via the Resistant Starch Assay Kit from Megazyme (Bray, Ireland) and was determined to be ∼20% for both the RS3_Ex and RS3_Wh.

All resistant starches were prepared via a pre-digestion step to simulate passage through the small intestine (treatment with pancreatin and amyloglucosidase), followed by non-thermal sterilization. Briefly, 2 g of sample were incubated at 37°C for 16 h in a shaking water bath with 800 mg porcine pancreatin and 900 μL amyloglucosidase (both from Sigma-Aldrich). Samples were rinsed 10 times with ∼30 mL DI water. Supernatant was removed by centrifugation at 3,000 × *g* for 5 min. Samples were sterilized overnight for ∼16 h with 70% ethanol. Samples were then rinsed 10 times with ∼30 mL of sterilized water. After the last rinse, samples were diluted to 2% (w/v) starch/sterile water suspensions. RS solutions were allowed to sit overnight in the anaerobic chamber before use to remove oxygen. CS was subjected to the same sterilization procedure without the pre-digestion step and Ap was sterilized via autoclave without pre-digestion.

### Other Materials

Digestive enzymes and buffer components were purchased through Sigma-Aldrich. Media components, HPLC consumables and DNA-polymerase were purchased through Thermo Fisher Scientific (Waltham, MA, United States). DNA extraction kits were purchased from Qiagen (Germantown, MD, United States). Sequencing supplies were from Illumina (San Diego, CA, United States).

### Microbial Strains

The resistant starch degrading bacteria *Bifidobacterium adolescentis* (ATCC 27255) and *Ruminococcus bromii* (L2-63) were grown in pure cultures prior to the beginning of fermentations so that they could be supplemented into PS fermentations (PS_Ba and PS_Rb, respectively) to test the impact of adding additional RS degrading organisms to these fermentations. This allows us to test if the numbers of these organisms are a limiting factor in the fermentation. The pure cultures were grown overnight in RUM media containing amylopectin (see below for media composition) to ensure expression of their starch degrading genes. The overnight cultures were diluted with sterile-reduced PBS to an optical density at 650 nm (OD_650_) of 1.5 and 50 μL of the culture was added to a 4 mL fermentation for a starting OD650 of ∼0.02 in these fermentations.

### Fecal Samples Used as Microbiome Inocula

Fecal samples were obtained through a concurrent study, examining the impact of potato and wheat dishes on cardiovascular markers and the gut microbiome, approved by the Penn State IRB (STUDY00007854). Subjects were between the ages of 25–75 years with BMI between 20 and 40 kg/m2. Subjects were non-smokers, and were free from metabolic and inflammatory diseases such as diabetes, hypertension and colitis. Samples were collected in sterile containers and immediately frozen at the subject’s home. Samples were transported to Penn State on ice within 24 h of collection and further stored at −80°C until needed. The study treatments were found to have only a modest impact on the microbiomes of the participants (unpublished results). The cardiovascular impacts of the treatments have been published (Johnston et al., 2020).

### Fermentation Experiments

The RUM media used for these experiments was originally developed (Ze et al., 2015) to support the growth of resistant starch degrading bacteria such as *R. bromii*. Before use, fecal samples were thawed under anaerobic conditions and diluted 1:3 w/v in pre-reduced PBS. For each inoculum/treatment combination under investigation, 200 μL of diluted fecal slurry was inoculated in triplicate into test tubes with the RUM media supplemented with either a treatment carbohydrate at 1% w/v or a no carbohydrate control (water), for a final volume of 4 mL. In total 11 different fecal inoculum sources (T01-T11) were investigated with a water control, two non-resistant starches (Ap and CS) and eight RS sources (Bn, HAM2, HAM4, PS, RS3_Ex, RS3_Wh, Tap, and Tn). Additionally, fermentations were carried out with PS supplemented with of *B. adolescentis* or *R. bromii* (PS_Ba and PS_Rb) as described above. Thus, a total of 13 treatment conditions were used. Note that RS3_Ex and RS3_Wh fermentations were not carried out with T10 and T11 due to an inadequate amount of material. After inoculation, samples were allowed to ferment for 24 h at 37°C, to simulate a colonic fermentation. Samples were then removed from anaerobic conditions, and their pH, fermentation profile, and final community structure were measured, as described below.

### Determination of Organic Acids and pH

To determine the fermentation profile produced from each experiment, samples were analyzed via high performance liquid chromatography (HPLC). The protocol used was derived from a previous study (Venkataraman et al., 2016) with some modifications. Briefly, one mL of each fermentation was removed and subjected to centrifugation at 16,010 × *g* for 10 min. The supernatant was filtered at 0.2 μm then diluted in 1:1 in 10 mM H_2_SO_4_ for a final concentration of 5 mM H_2_SO_4_. Thermo Fischer’s Dionex 5000+ series HPLC was used in this experiment, with a 50 mm guard column (Micro-Guard Cation H Cartridge, Bio Rad, Hercules, CA, United States) and a 300 mm ion exclusion column (Aminex HPX-87H, Bio-Rad, Hercules, CA, United States). Runs were 60 min long at 50°C, 4.5 mL/min flow rate, with no temperature or flow gradients. Organic acids were detected by UV absorbance at 214 nm. Quantification was done with a standard curve of each of acetate, butyrate, formate, lactate and succinate and subtraction of a media blank. It was determined that propionate could not be accurately determined due to co-elution of a media component that largely obscured the signal and could not be separated from propionate without resorting to prohibitively long run times. Differences in organic acid levels were measured between each of the starch containing treatments and the no-starch control. Organic acid data was found to follow a skewed distribution and was subjected to a ln(x+1) transformation to normalize the data prior to statistical testing. Changes in each organic acid for each treatment were analyzed with a linear mixed effects model with inoculum source as a random effect using the lmer function of the lmerTest package (Kuznetsova et al., 2017) in R. *P*-values were corrected via the Benjamini-Hochberg FDR procedure (Benjamini and Hochberg, 1995).

The pH was measured on a Denver Instrument (Denver, CO, United States) Model 250 pH and conductivity meter. Changes in pH were tested as described for the organic acids but without prior transformation.

### Microbiome Analysis

#### 16S Amplicon Sequencing

DNA was extracted from one random replicate of each treatment. Qiagen’s Power Fecal DNA Kit was used, and protocol followed according to the manufacturer’s instructions, except that bead beating was carried out on a BeadBeater96 (Biospec, Bartlesville, OK, United States) for 5 min at 3,800 rpm. Extracted DNA was diluted 1:10 in sterile water and stored frozen at −20°C before PCR and sequencing. PCR was used to amplify the 16S rRNA V4 region and purity was confirmed with gel electrophoresis. The forward primer used was 505F (5′-TCG TCG GCA GCG TCA GAT GTG TAT AAG AGA CAG GTG YCA GCM GCC GCG GA A-3′) and reverse primer 806R (5′-GTC TCG TGG GCT CGG AGA TCT GRA TAA GAG ACA GGG ACT ACN VGG GTW TCT AAT-3′). PCR run conditions were as follows: 94°C for 2 min, 94°C for 20 s, 56°C for 30 s, 68°C for 40 s, repeat for 30 cycles, then 72°C for 5 min. PCR products were sent to Penn State’s Genomics Core Facility where a second round of PCR was performed to add Illumina adapters. Library quality control was performed on an Agilent Bioanalyzer and confirmed PCR products were normalized by concentration and purified before running on an Illumina MiSeq using v2 reagent kits and 250 × 250 nucleotide paired-end sequencing.

Scripts used for data processing can be found at: https://github.com/darrell25/Teichmann_2020. De-multiplexed sequences were returned from the sequencing core and primer sequences were removed with the program cutadapt (v3.1) (Martin, 2011). Quality control filtering of sequencing reads was performed in the program mothur (Schloss et al., 2009), utilizing the MiSeq SOP method^1^ accessed October 6, 2020. Forward and reverse reads were merged and reads of incorrect length or with ambiguous reads were screened out. Unique reads were then aligned to version 132 of the Silva Database (Quast et al., 2013). Chimeric sequences were removed with UCHIME (Edgar et al., 2011) and de novo OTUs were generated using the opticlust algorithm at a cutoff of 0.03 or 97% similarity. Genus level taxonomic assignment was carried out with the RDP classifier and version 18 of the RDP training set (Cole et al., 2014). Blast+ (Camacho et al., 2009) was used to assign species level taxonomy using the representative sequence of each OTU. Species names were only assigned if the BLAST hit had at least 97% identity, otherwise the genus level designation for the OTU from RDP was used. A phylogenetic tree for the OTUs was produced using the program FastTree (Price et al., 2010) with a representative FASTA sequence for each OTU.

#### Diversity Analysis

Alpha diversity and beta diversity were analyzed at the genus level in R using the phyloseq package (McMurdie and Holmes, 2013) and other packages that make use of the phyloseq architecture. For all analyses a phyloseq object was first created consisting of a transposed version of the OTU table (shared file) generated by mothur (Schloss et al., 2009), the taxonomy table generated by mothur updated with Blast+ (Camacho et al., 2009) species identifications and formatted for phyloseq, the phylogenetic tree produced by FastTree (Price et al., 2010) and a meta data file containing inoculum IDs and treatments as factors. The tax_glom function of phyloseq (McMurdie and Holmes, 2013) was used to combine OTUs at the genus level.

For alpha diversity, Faith’s phylogenetic diversity, Shannon diversity and inverse Simpson diversity were analyzed. For Faith’s phylogenetic diversity the OTU count data was first rarefied to an even number of reads for each sample by random sub-sampling to 2490 reads (the lowest number in any sample). Diversity was then calculated with the pd function of the picante package (Kembel et al., 2010). The resulting diversity measures were then compared between treatments with a linear mixed effect model with inoculum source as a random effect using the lmer function of the lmerTest package (Kuznetsova et al., 2017). For Shannon and inverse Simpson diversity the counts were not rarefied and were instead processed via the DivNet package (Willis and Martin, 2020) which also generated confidence intervals for each measurement. These were then analyzed for significant differences between each treatment and the control with the betta_random function of the breakaway package (Willis et al., 2017) using fecal inoculum as a random effect.

For beta diversity, weighted UniFrac, Bray-Curtis distance, and Aitchison distance analyses were performed. For weighted UniFrac analysis the data were rarefied to an even number of reads for each sample by random sub-sampling. The analysis was performed with the UniFrac function of the phyloseq package. Bray-Curtis dissimilarities were generated with the DivNet package concurrently with the alpha diversity measures. Aitchison distances were calculated by performing a centered-log-ratio transformation of the data with the Microbiome package^2^, followed by the calculation of Euclidian distance via the Vegan package^3^. All beta diversity distance or dissimilarity matrices were ordinated via principle coordinate analysis and plotted to look for treatment-based clustering. Treatment-driven differences in beta diversity were statistically tested via PERMANOVA using the adonis function (Anderson, 2001) in the Vegan package. There is currently no method available to account for random effects in beta diversity analysis. Individual treatment-control differences were implemented via the calc_pairwise_permanovas function of the mctoolsr package^4^.

#### Differential Abundance Analysis

Differential abundance analysis comparing each of the starch treatments to the no-starch control was performed with three techniques with differing statistical approaches: LEfSe (Segata et al., 2011), DESeq2 (Love et al., 2014), and ANCOM-II (Kaul et al., 2017). In all cases the taxa were first filtered for abundance (minimum 0.001% of total reads) and prevalence (present in at least 5% of samples). For LEfSe reads were first converted to relative abundances at each taxonomic level. LEfSe was run with the default settings through the Galaxy web application from the Huttenhower Lab^5^, with fecal inocula coded as ‘‘individuals.’’ DESeq2 was run with the default parameters in the DESeq2 package in R. ANCOM-II analysis was performed using the R implementation of Huang Lin^6^. Default parameters were used except that the zero_cut parameter (1 – prevalence fraction) was adjusted to 0.95 to match the analysis performed with the other methods. Taxa were considered differentially abundant if they passed the 0.7 threshold of the method (recommended setting). For all methods, *P*-values were corrected with the Benjamini-Hochberg method (Benjamini and Hochberg, 1995). In all cases analysis was performed at the phylum, genus and species levels, condensing taxonomic levels as needed with the tax_glom function of phyloseq.

### Correlation Analysis

Spearman correlations were examined between butyrate and each of the other organic acids as well as pH. Tests and plots were generated via functions in the ggpubr package^7^. All *P*-values were corrected with the Benjamini-Hochberg method (Benjamini and Hochberg, 1995).

For correlations between key microbiome members, the first 200 OTUs were examined for known butyrate producing and RS degrading members of the microbiome. For RS degraders this was limited to OTUs identified as either *Bifidobacterium adolescentis* or *Ruminococcus bromii* (no other RS degrading bifidobacteria were detected within the first 200 OTUs). Butyrate producers were limited to those known to produce butyrate through the acetyl-CoA pathway for butyrate from carbohydrates. This included organisms previously reported to produce butyrate from carbohydrates through direct tests (Louis and Flint, 2017) as well as those determined to have the genomic potential for producing butyrate from carbohydrates (Vital et al., 2014), see Supplementary Table 2. The OTU count data was first subjected to centered-log-ratio transformation in the context of the full dataset prior to sub-setting to the butyrate producing and RS degrading taxa. Insufficient replications were available to reliably test correlations for each treatment individually, so instead treatments were divided into two groups; a low butyrate group where butyrate production was equal to or lower than in the control and a high butyrate group where butyrate production was greater than in the control. Spearman correlations were then calculated between the main *R. bromii* OTU, the main *B. adolescentis* OTU, butyrate levels and the pH with the key taxa in each of these treatment groups utilizing the microbiomeSeq package^8^. All *P*-values were corrected with the Benjamini-Hochberg method (Benjamini and Hochberg, 1995).

## Results

### Changes in Organic Acid Production and pH Across Treatments and Inocula

**FIGURE 1:**
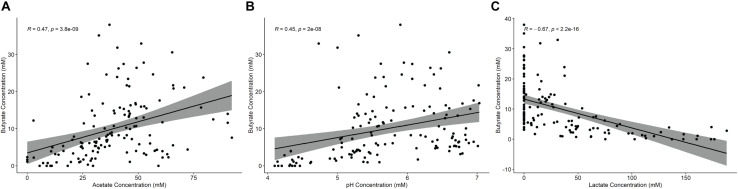
Correlation analysis of fermentation parameters with butyrate production. Spearman correlations of butyrate concentrations with **(A)** acetate, **(B)** pH, or **(C)** lactate across all treatments. *P-*values are corrected by FDR.

Fermentations were conducted with 11 different fecal inocula and a total of 12 different starch supplementations, 10 of which were RS sources. Comparing across these treatments (Table 1) the pH dropped significantly in most cases relative to the starch-free control, with the exception of the tiger nut flour (Tn) supplementation. This seemed to be driven largely by lactate production which increased substantially in most of the fermentation conditions except for banana flour (Bn), high amylose maize starch (HAM2) and Tn. The non-resistant starches amylopectin (Ap) and corn starch (CS) along with the extracted, retrograded, potato starch (RS3_Ex) experienced both the largest pH drops and the largest increases in lactate concentration. The other fermentations seemed to stay in a pH range that is more representative of the colon, which has been found to vary between approximately pH 5 and 8 (Koziolek et al., 2015). Butyrate production decreased significantly from the control in both the Ap and CS conditions and was only found to increase significantly in the Bn condition. Formate levels did not change significantly in any of the treatments, while succinate increased significantly only in Ap and acetate decreased significantly only in Ap (Supplementary Table 3). Overall, butyrate levels were found to be positively correlated with both pH and acetate and negatively correlated with lactate levels (Figure 1). The opposite trends existing for acetate and lactate are interesting since both have pathways in the microbiome for conversion to butyrate (Duncan et al., 2002; Belenguer et al., 2006).

**TABLE 1:** Differences in fermentation parameters between treatments and control averaged across all inocula.

Starch^a^	pH			Butyrate (mM)			Lactate (mM)		
	Value	Δ^b^	*P*^c^	Value	Δ	*P*	Value	Δ	*P*
Ap	4.4 ± 0.3	−2.4	***<0.001***	4.3 ± 9.3	−4.4	***<0.001***	127 ± 54	126	***<0.001***
CS	5.0 ± 0.6	−1.8	***<0.001***	9.8 ± 13	1.1	0.756	71 ± 59	70	***<0.001***
Bn	6.2 ± 0.4	−0.6	***0.002***	18 ± 5.6	9.3	***0.022***	1.6	1.0	0.727
Tn	6.8 ± 0.2	−0.0	0.793	11 ± 5.9	2.3	0.588	0.0 ± 0.0	−0.6	0.727
HAM2	6.3 ± 0.4	−0.5	***0.008***	11 ± 7.3	2.3	0.955	4.3 ± 6.6	3.7	0.151
PS	5.8 ± 0.5	−1.0	***<0.001***	10 ± 7.8	1.3	0.994	16 ± 17	15	***<0.001***
PS_Ba	5.4 ± 0.4	−1.4	***<0.001***	7.0 ± 6.5	−1.7	0.673	27 ± 18	26	***<0.001***
PS_Rb	5.6 ± 0.3	−1.2	***<0.001***	9.7 ± 7.2	1.0	0.994	28 ± 24	27	***<0.001***
RS3_Ex	4.9 ± 0.6	−1.9	***<0.001***	6.8 ± 8.4	−1.9	***0.022***	82 ± 55	81	***<0.001***
RS3_Wh	5.3 ± 0.6	−1.5	***<0.001***	12 ± 12	3.3	0.994	38 ± 28	37	***<0.001***
HAM4	5.7 ± 0.5	−1.1	***<0.001***	11 ± 9.6	2.3	0.994	32 ± 33	31	***<0.001***
Tap	5.6 ± 0.5	−1.2	***<0.001***	10 ± 7.7	1.3	0.994	32 ± 33	31	***<0.001***
Water	6.8 ± 0.1	0		8.7 ± 4.8	0		0.6 ± 2.0	0	

*^a^Abbreviations are: Ap, amylopectin; CS, corn starch; Bn, green banana flour; Tn, tiger nut flour; HAM2, high amylose maize starch; PS, potato starch; PS_Ba, potato starch with fecal samples supplemented with Bifidobacterium adolescentis; PS_Rb, potato starch with fecal samples supplemented with Ruminococcus bromii; RS3_Ex, retrograded extracted potato starch; RS3_Wh, retrograded whole potatoes; HAM4, chemically cross-linked high amylose maize starch; Tap, chemically cross-linked tapioca starch; Water, no starch control. ^b^Change relative to the no-starch control (Water).^c^Significant P values (P < 0.05) are indicated by bolded/italicized values.*

**FIGURE 2:**
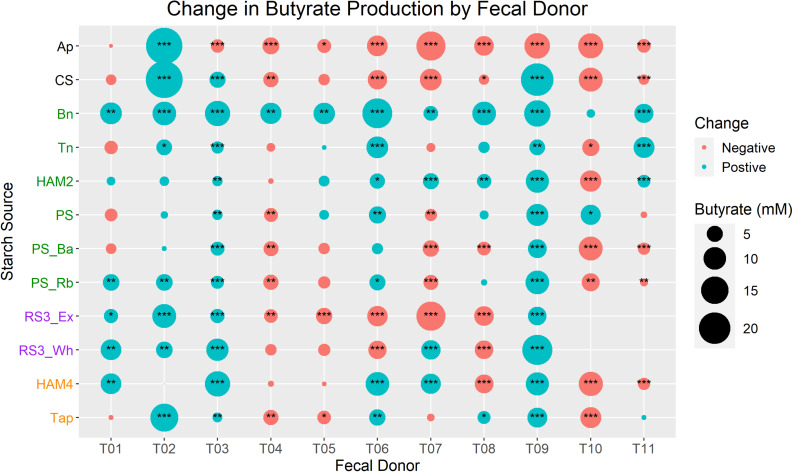
Change in butyrate production between control and treatments by fecal donor. In each case butyrate production during the control fermentation for a given inoculum is subtracted from the butyrate production in a given treatment with that same inoculum. Bubble size is proportional to the total change in butyrate production, colored red for a decrease and blue for an increase. *Y*-axis labels of starch sources are colored by RS designation. Black is non-RS, green is RS2, purple is RS3, orange is RS4. Statistical significance of the difference is calculated by one-way ANOVA of the ln(x+1) transformed butyrate values and FDR correction of *P*-values. Labels are *P < 0.05, **P < 0.01, ***P < 0.001. Ap, amylopectin; CS, corn starch; Bn, green banana flour; Tn, tiger nut flour; HAM2, high amylose maize starch; PS, potato starch; PS_Ba, potato starch with fecal samples supplemented with *Bifidobacterium adolescentis*; PS_Rb, potato starch with fecal samples supplemented with *Ruminococcus bromii*; RS3_Ex, retrograded extracted potato starch; RS3_Wh, retrograded whole potatoes; HAM4, chemically cross-linked high amylose maize starch; Tap, chemically cross-linked tapioca starch.

When examining changes in butyrate production for each inoculum/treatment combination, significant inter-individual differences arise (Figure 2). Only the Bn treatment caused a near universal increase in butyrate production as only the increase for T10 was not significant. All other treatments had some inocula for which they significantly increased butyrate, but they were far less universal. The Ap treatment only significantly increased butyrate production for the T02 inoculum, though interestingly this was one of the largest butyrate increases seen. Neither T01 nor T02 had any conditions in which there was a significant decrease in butyrate relative to the control, but they also had some of the lowest control fermentation butyrate levels. The T04, T05, and T10 inocula each only had one treatment that significantly increased butyrate production, Bn in the case of T04 and T05 and potato starch (PS) in the case of T10. Interestingly the T04 and T05 inocula came from the same donor several weeks apart, showing a remarkably consistent profile of fermentation results. The T03 and T09 inocula had the most consistent increases in butyrate, increasing significantly for all treatments except for Ap. These patterns of increase were similar for T03 and T09, though they were at opposite ends of the spectrum in the control fermentation with T03 producing 3.3 mM, the lowest of any of the inocula, while T09 produced these large gains despite having the highest control fermentation levels of butyrate at 16.9 mM. Intriguingly T09 is the only inoculum that completely lacks *B. adolescentis*, ensuring that the RS degradation is completely dominated by *R. bromii*.

### Changes in Diversity With Starch Treatment

**FIGURE 3:**
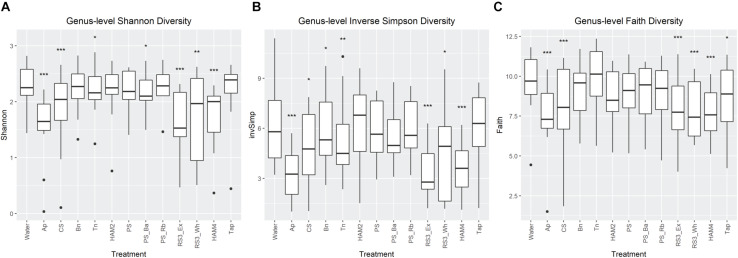
Comparison of genus-level alpha diversity metrics between treatments. Shannon diversity **(A)** and inverse Simpson diversity **(B)** and their significant differences between the treatments no-carbohydrate added control (labeled as Water) were calculated via the DivNet package in R. Faith’s Phylogenetic Diversity **(C)** was calculated via the Picante package in R and significant differences determined via a linear mixed effects model. All significant differences were calculated as comparisons to the control with FDR correction. Labels are *P < 0.05, **P < 0.01, ***P < 0.001. Ap, amylopectin; CS, corn starch; Bn, green banana flour; Tn, tiger nut flour; HAM2, high amylose maize starch; PS, potato starch; PS_Ba, potato starch with fecal samples supplemented with *Bifidobacterium adolescentis*; PS_Rb, potato starch with fecal samples supplemented with *Ruminococcus bromii*; RS3_Ex, retrograded extracted potato starch; RS3_Wh, retrograded whole potatoes; HAM4, chemically cross-linked high amylose maize starch; Tap, chemically cross-linked tapioca starch.

Genus level alpha diversity was analyzed across each of the treatments (Figure 3). Three diversity measures were utilized, Shannon diversity, Inverse Simpson diversity and Faith’s phylogenetic diversity. Shannon diversity places more weight on richness, Inverse Simpson places more weight on evenness of the OTU distribution and Faith’s considers how related the differing OTUs are to one another. All three metrics showed a general trend of decreasing diversity relative to the control fermentation. This is unsurprising as the supplemented starches are selective for the bacteria that can effectively use starch. Indeed, feeding trials with RS have generally shown decreased diversity upon RS supplementation (Bendiks et al., 2020). For all indices it was the Ap, RS3_ex, and the chemically modified high amylose maize starch (HAM4) that showed the greatest drop in diversity. The first two are unsurprising as these were the fermentations that experienced the greatest pH drop (Table 1), which likely had a negative impact on diversity. However, the nearly equal drop in diversity for HAM4 is more surprising as it had only an average pH drop, suggesting other mechanisms were at play.

**FIGURE 4:**
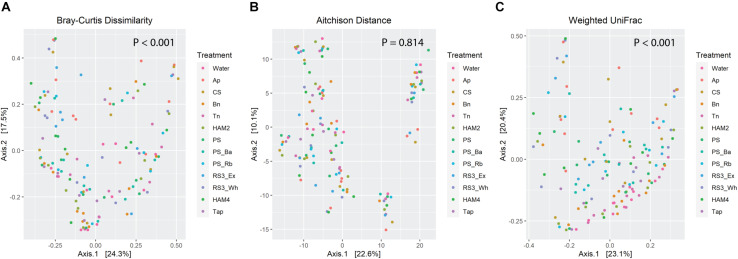
Principle coordinate analysis plots of beta diversity metrics, with points colored by treatment. *P*-values are calculated by PERMANOVA via the Adonis function in the Vegan package in R. **(A)** Bray-Curtis dissimilarities derived from the DivNet package in R. **(B)** Aitchison distances calculated as the Euclidean distance between microbiomes following centered-log-ratio transformation of the count data, calculated via the Vegan and Microbiome R packages. **(C)** Weighted UniFrac distance calculated using the Phyloseq package in R on rarefied count data.

Beta diversity was also examined through three metrics, Bray-Curtis, Aitchison and weighted UniFrac (Figure 4). Some degree of clustering is apparent in the PCoA plots of all three metrics, though the treatment related effects are not immediately obvious. Nevertheless, PERMANOVA analysis of the Bray-Curtis results and weighted UniFrac show significant treatment-based separation of the microbiomes. A pairwise PERMANOVA explicitly comparing differences between each treatment and the control (Table 2), shows significant differences between the Ap and control as well as the RS3_Ex and control for all three metrics. The CS, HAM4, PS_Ba, PS_Rb, and retrograded whole potatoes (RS3_Wh) show significant differences for both Bray-Curtis and weighted UniFrac, while chemically modified tapioca starch (Tap) is only significantly different via weighted UniFrac.

**TABLE 2:** PERMANOVA results comparing beta diversity metrics between treatments and control.

Starch^a^	*P* Bray-Curtis^b^	*P* Aitchison	*P* Weighted UniFrac
Ap	***0.002***	***0.012***	***0.002***
CS	***0.002***	0.382	***0.002***
Bn	0.735	0.991	0.561
Tn	0.968	0.991	0.892
HAM2	0.185	0.991	0.057
PS	0.204	0.991	0.089
PS_Ba	***0.002***	0.812	***0.002***
PS_Rb	***0.017***	0.944	***0.009***
RS3_Ex	***0.002***	***0.024***	***0.002***
RS3_Wh	***0.002***	0.132	***0.002***
HAM4	***0.004***	0.382	***0.002***
Tap	0.068	0.944	***0.029***

*^a^Abbreviations are: Ap, amylopectin; CS, corn starch; Bn, green banana flour; Tn, tiger nut flour; HAM2, high amylose maize starch; PS, potato starch; PS_Ba, potato starch with fecal samples supplemented with Bifidobacterium adolescentis; PS_Rb, potato starch with fecal samples supplemented with Ruminococcus bromii; RS3_Ex, retrograded extracted potato starch; RS3_Wh, retrograded whole potatoes; HAM4, chemically cross-linked high amylose maize starch; Tap, chemically cross-linked tapioca starch; Water, no starch control.^b^Significant P values (P < 0.05) are indicated by bolded/italicized values.*

### Changes in Taxa Across Starch Treatments

**FIGURE 5:**
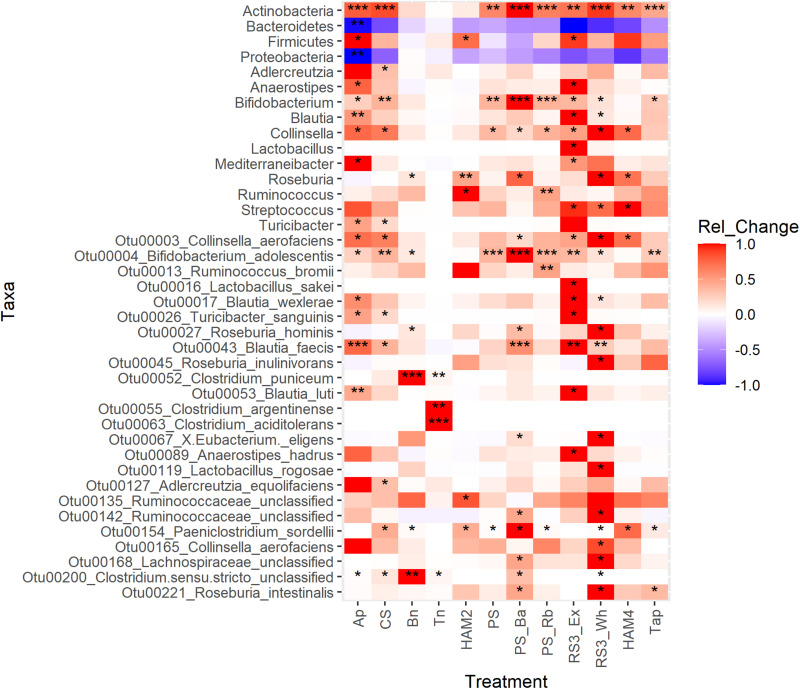
Differential abundance analysis heatmap of taxonomic features between treatments. The heatmap is colored by the standardized change in the relative abundance of the taxon between the treatment and the control. Red indicates a positive change; blue indicates a negative change. Significant differences were calculated by three methods: LEfSe, DESeq2 and ANCOM-II. Significance is indicated by * for one method finding the taxon significantly different between that treatment and the control, ** for two methods detecting significant difference and *** for all three methods detecting a significant change. For LEfSe and DESeq2, adjusted *P*-values of 0.05 were used as the cutoff for significance, while for ANCOM-II which does not generate *P*-values, the default cutoff threshold of 0.7 was used. Ap, amylopectin; CS, corn starch; Bn, green banana flour; Tn, tiger nut flour; HAM2, high amylose maize starch; PS, potato starch; PS_Ba, potato starch with fecal samples supplemented with *Bifidobacterium adolescentis*; PS_Rb, potato starch with fecal samples supplemented with *Ruminococcus bromii*; RS3_Ex, retrograded extracted potato starch; RS3_Wh, retrograded whole potatoes; HAM4, chemically cross-linked high amylose maize starch; Tap, chemically cross-linked tapioca starch.

**FIGURE 6:**
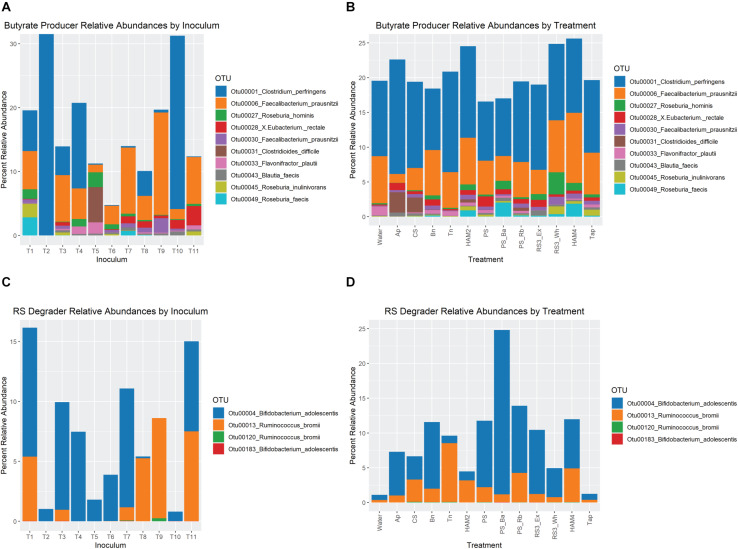
Relative abundance of butyrate producing and resistant starch degrading organisms. **(A)** Relative abundance of OTUs associated with butyrate producing organisms at the end of fermentation, averaged for each starting inoculum across all treatments. **(B)** Relative abundance of OTUs associated with butyrate producing at the end of fermentation, averaged for each treatment across all inocula. For both **(A,B)** only the top 10 most abundant butyrate-producer OTUs are included. **(C)** Relative abundance of OTUs associated with resistant starch degrading organisms at the end of fermentation, averaged for each starting inoculum across all treatments. **(D)** Relative abundance of OTUs associated with resistant starch degrading organisms at the end of fermentation, averaged for each treatment across all inocula. Ap, amylopectin; CS, corn starch; Bn, green banana flour; Tn, tiger nut flour; HAM2, high amylose maize starch; PS, potato starch; PS_Ba, potato starch with fecal samples supplemented with *Bifidobacterium adolescentis*; PS_Rb, potato starch with fecal samples supplemented with *Ruminococcus bromii*; RS3_Ex, retrograded extracted potato starch; RS3_Wh, retrograded whole potatoes; HAM4, chemically cross-linked high amylose maize starch; Tap, chemically cross-linked tapioca starch.

Differential abundance analysis was performed between the treatments and control using three methods LEfSe, DESeq2, and ANCOM-II. Each uses a different approach to overcome the challenges of analyzing microbiome data and the inability to know the true abundances of the OTUs under investigation and thus agreement of two or more of these methods in identifying differential taxa can increase confidence in the results. A heatmap is shown in Figure 5, where the number of stars indicates the number of different methods that found the taxon to be significantly different between that treatment and the control. Other than at the phylum level, only taxa that increased in one of the treatments are shown. Those that decreased significantly were almost universally members of the Bacteroidetes or the Proteobacteria phyla and are not depicted. The most consistently increasing bacterium was *B. adolescentis*, unsurprising as a key RS degrading microbe. More surprisingly the other main RS degrading bacterium *R. bromii* was only found to be significantly increased versus the control in the PS_Rb condition where it had been supplemented in at the start of the fermentation. Even in this condition *B. adolescentis* was the main RS degrading organism during the fermentation (Figure 6D), illustrating the strong competitive advantage *B. adolescentis* has over *R. bromii* on PS. The only conditions where *R. bromii* outnumbered *B. adolescentis* were the structurally related HAM2 and HAM4 and these were the only conditions that *B. adolescentis* did not significantly increase under. Beyond the RS degraders, the butyrate producer *Blautia faecis* was significantly increased in Ap, CS, PS_Ba, RS3_Ex, and RS3_Wh. *Clostridium puniceum* was the most strongly differential species in the Bn condition, which was the most consistent butyrate increasing condition (Figure 2), however, *C. puniceum* is not a butyrate producing organism. The *Roseburia* genus and particularly *R. hominis*, was the only butyrate producing group found to have significantly increased in the Bn condition, though it was only detected by LEfSe. The *Roseburia* genus was also found to be increased in HAM2, HAM4, PS_Ba, and RS3_Wh, but these were only detected via LEfSe apart from HAM2 which was also detected via DESeq2. *Clostridium argentinense* and *Clostridium aciditolerans* were both only found in the Tn condition, suggesting that they were contaminants present in the Tn flour itself and survived the sterilization treatment. Several other taxa were found to be differential by one of these methods and may be important, but there is less certainty with these results.

Looking at the relative abundances of the most prevalent butyrate producing organisms and RS degrading organisms shows much more dramatic interindividual differences than across treatments (Figures 6A–D). For instance, inoculum T09 is the only one where *B. adolescentis* is completely absent. *R. bromii* is absent from T02, T04, T05, T06, and T10. Note that the T04 and T05 inocula come from the same individual. However, looking across treatments there are more stable ratios between *R. bromii* and *B. adolescentis*, with *B. adolescentis* dominating under most conditions, with the exception of HAM2 and HAM4. Despite T09 and T03 exhibiting a similar pattern of butyrate increase across treatments (Figure 2), there is little similarity in their microbial composition with regards to RS degraders or butyrate producers. However, it should be noted that while the pattern of increase was similar, the magnitude of butyrate production was much different. The highest butyrate production for T03 was 16 mM with Bn, which was lower than even the control fermentation for T09 at 16.9 mM. Among the butyrate producers, inocula T10 and T02 are largely dominated by *Clostridium perfringens*, representing the only detected butyrate producer in T02, suggesting that this is a particularly anomalous sample. The expansion of *Clostridoides difficile* in Ap and CS treatments seems to be entirely due to inoculum T05 where levels are particularly high. More subtle differences in the relative abundances of butyrate producers are evident across treatments, but as can be seen in Figure 5, most did not reach the level of statistical significance once estimates of true abundances were considered.

### Correlations Between Key Taxa and Butyrate Production

**FIGURE 7:**
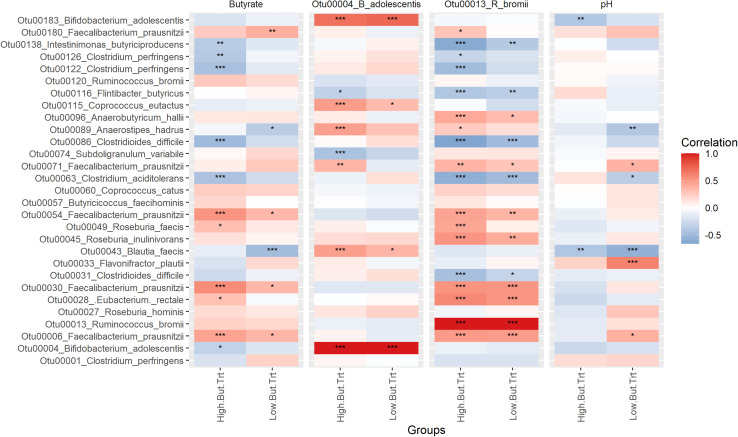
Spearman correlations between fermentation features, resistant starch degrading organisms and butyrate-producing organisms. Centered-log-ratio transformed counts of bacterial taxa were tested for Spearman correlation with similarly transformed counts of resistant starch degrading taxa, butyrate concentrations and pH. The heatmap is colored by correlation coefficient. Significance levels are the FDR-adjusted *p*-values across each panel. *P < 0.05, **P < 0.01, ***P < 0.001. Panels for each comparison are divided into the low butyrate producing treatments (water control, amylopectin, corn starch, extracted retrograded potato starch, retrograded whole potato and tiger nut flour) and the high butyrate producing treatments (banana flour, high amylose maize starch, chemically cross-linked high amylose maize starch, potato starch, potato starch with fecal samples supplemented with *Bifidobacterium adolescents*, potato starch with fecal samples supplemented with *Ruminococcus bromii* and chemically cross-linked tapioca starch). The low butyrate treatments are those producing equal to or less butyrate than in the water control, whereas the high butyrate treatments are those that produce more butyrate than in the control fermentation.

Perhaps more interesting than what is differential between the treatments is which OTUs are driving butyrate production. Examining correlations between butyrate production, RS degrading taxa and butyrate producing taxa (Figure 7) reveals that certain taxa are more important drivers than others. In this analysis treatments are grouped as either high butyrate producing (Bn, HAM2, HAM4, PS, RS3_Wh, Tap, and Tn) or low butyrate producing (Water, Ap, CS, PS_Ba, PS_Rb RS3_Ex, and Tn), using average production of 10 mM butyrate as the cutoff (Table 1). There are too few replicates to allow for reliable correlation analysis for each treatment individually. The *Faecalibacterium prausnitzii* OTUs (Otu00006, Otu00030, and Otu00054) were the most strongly positively correlated with butyrate, particularly for the high butyrate treatments. *Eubacterium rectale* and *Roseburia faecis* were also positively correlated in the high butyrate treatments while the Otu00180 *F. prausnitzii* was also positively correlated in the low butyrate conditions. Neither of the RS degraders were positively correlated with butyrate levels, however, there were a number of strong correlations between the RS degraders and the butyrate producing organisms. *R. bromii* had strong positive correlations with *E. rectale*, *R. faecis*, *R. inulinivorans*, and the *F. prausnitzii* OTUs and generally strong negative correlations with members of the Clostridiaceae and *C. difficile*. In contrast *B. adolescentis* had fewer connections to the butyrate producers, though with strong positive correlations with *Coprococcus eutactus*, *Anaerostipes hadrus*, *Blautia faecis*, and the Otu00071 *F. prausnitzii*, mostly in the high butyrate producing treatments. The relationship with *A. hadrus* may be particularly important as it along with *Coprococcus catus* and *Anaerobutyricum hallii* are key taxa that are able to convert lactate that is the main fermentation product of *B. adolescentis* to butyrate (Duncan et al., 2004). The correlations between pH and butyrate producers were generally less significant, though stronger in the low butyrate conditions. Overall, although *R. bromii* does not directly produce butyrate and is not strongly correlated to butyrate levels itself it is a very important determinant of the levels of butyrate producing organisms, though other factors may impact how much butyrate they actually produce.

## Discussion

### Media Used for Fermentation Experiments

In conducting these experiments we have utilized the RUM media (Ze et al., 2015) developed for growth experiments with *R. bromii*, a relatively fastidious organism, but also a key player in RS utilization in the gut (Ze et al., 2012). Media that have been used in recent studies of *in vitro* fermentations of gut communities can be divided into three broad categories. These are, with some examples, (1) rich undefined media such as brain heart infusion that provide fairly complete nutritional sources in an attempt to insure all possible requirements are present (Chen Y. et al., 2020), (2) defined or semi-defined media that attempt to recapitulate most of the key components of rich undefined media, but in a more controlled way (Williams et al., 2005; Gu et al., 2018; Feng et al., 2020) and (3) defined or semi-defined media that provide key basic nutrients, but rely on the community of microbes to produce the majority of the more complex vitamin requirements through a cross-feeding network (Li et al., 2019; Gerasimidis et al., 2020). The RUM media that we have used here falls into this second category, somewhat mimicking rumen fluid, which has in the past been a useful undefined source of nutrients that has been successfully used to culture otherwise recalcitrant anaerobic microbes. The third approach most closely mimics what natively occurs in the gut environment, but the risk exists in short-term batch fermentations that factors such as required vitamins will not reach sufficient levels to adequately support organisms such as *R. bromii* and other important organisms with complex growth requirements. This is less of a concern in continuous fermentation systems (McDonald et al., 2013; Robinson et al., 2014; Poeker et al., 2018) where an equilibrium is reached over time and these components can be adequately supplied by other microbes. Recently Li et al. (2019) developed the MiPro media, which fall under the third approach listed above, and validated its ability to sustain a community that from a metagenomics and metaproteomics perspective appears to closely mimic the fecal community from which it was derived. This is an important consideration when developing an *in vitro* fermentation system, however, it may not be a one size fits all approach, particularly when there are key taxa for a phenomenon under study that may not be adequately supported. In the MiPro study the abundance of *R. bromii* was not particularly noted, though another key fastidious organism in the gut, *F. prausnitzii* was found to decrease in relative abundance in the early stages of fermentation before stabilizing, perhaps due to the need for a buildup of required nutrients. Besides containing few added vitamins, the MiPro media differs from the RUM media by including mucins as a basal carbohydrate and peptide source and bile salts as a natural selective factor to help mimic the gut environment. We have experimented with both additions but found in each case that they interfered with our ability to analyze RS fermentations either by suppressing total fermentation (bile salts) or by providing a competing carbohydrate source (mucin). These are, however, important factors for mimicking the gut environment and future work should endeavor to incorporate them into *in vitro* fermentation experiments.

In terms of the utilization of a 24-h time point Li et al. found that similarity of the *in vitro* metagenome and metaproteome to those found *in vivo* peaked around 9–24 h, with decreases in the bacterial population after that point (Li et al., 2019). Furthermore 24 h is more or less consistent with residence times in the later sections of the colon, with total colon residence times averaging 30–40 h in healthy subjects (Arhan et al., 1981; Tomita et al., 2011). Thus, 24 h offers a reasonable compromise between biologically relevant fermentation and the limits of *in vitro* techniques. For insoluble and slowly digested fibers such as resistant starch, mimicking conditions in late colon is highly biologically relevant, whereas for other more quickly digested soluble fibers such as inulin (Fehlbaum et al., 2018) and shorter degree of polymerization prebiotics (Chen M. et al., 2020), the late small intestine and early colon would be the most relevant. The short time frame in these 24 h fermentations does not necessarily allow for full adaptation of microbes to their new conditions, for which a continuous culture system would be more appropriate to investigate (Poeker et al., 2018) but does mimic an acute change in diet as a new food source is provided to the microbiome.

### Microbiome Fermentation Capacity Variation Over Time

In this study most of the starting microbiomes came from different individuals, however, two samples came from the same individual at two timepoints several weeks apart (T04 and T05). Past studies have examined microbiome stability over time and found that despite daily variations due to diet (David et al., 2014), gut microbiomes are relatively stable over the adult years (Faith et al., 2013; Sinha et al., 2018). These analyses tend to look at the microbiome as a whole and may not account for more subtle changes in key taxa that might impact fermentative capacity. In the current study, despite changes in the relative abundances of butyrate producers and RS degraders (Figures 6A,C), the fermentation changes in response to a panel of starch sources was remarkably similar (Figure 2 and Supplementary Figures 1–4). This suggests that this property of a microbiome may be quite stable, though further investigation with more individuals is needed.

### Impact of Pure RS vs. Whole Flours vs. Non-RS

The Bn condition was the most consistently favorable for increases in butyrate production relative to the no-starch added control (Figure 2). It was also one of three added starch sources (along with Tn and RS3_Wh) where it was not simply a pure starch, but rather a high starch content flour, including other materials. This would potentially include other fibers that may act synergistically with the RS to produce more butyrate. While Tn was not as universally butyrogenic as Bn, it still produced a significant increase in butyrate for 5 out of 11 individuals. If we consider net frequency of butyrate increase (# of significant butyrate increases – # of significant butyrate decreases) then it was the third best performing condition at +4, following Bn at +10 and HAM2 at +5. The RS3_Wh was at +3 by this metric, but this is a notable increase over the best direct comparable to pure starch sources for these purposes, the RS3_Ex at −1. This suggests that there are possibilities for synergy between RS and other dietary fibers for promoting butyrate production in the gut. There may also be a certain degree of complementarity between RS sources, where for example the RS3_Wh and HAM2 might be expected to perform better in combination, given the relatively complementary nature of their butyrate profiles across individuals (Figure 2).

In considering the butyrate responses to these different forms of RS it is also interesting to consider the fermentations with non-RS sources Ap and CS. Typically the reason these starches are not thought to be major butyrate drivers in the gut is that they are degraded by the human enzymes and their sugars largely absorbed before they reach the colon. Interestingly in this case where that human enzyme step was essentially bypassed, they were still poor at eliciting increases in butyrate. The definition of RS centers on its resistance to degradation by human enzymes but it is likely that starches that are resistant to human enzymes are also resistant to most microbial enzymes (outside of the RS degraders). For instance, the cell-wall anchored amylase of *E. rectale* has very poor activity against PS, moderate activity against CS and very good activity against Ap (Cockburn et al., 2018). Thus, during these fermentations, it would have been able to directly utilize the CS and Ap for growth, but not the PS without the intervention of an RS degrader. *E. rectale* is a butyrate producer as are *Roseburia* species that have similar enzymes (Ramsay et al., 2006), so there is at least potential for butyrate increases with these substrates. It should be noted though that the GH13 class of enzymes that contains most starch degrading enzymes is nearly ubiquitous among gut organisms (El Kaoutari et al., 2013), suggesting that the ability to grow on starch and/or starch breakdown products is quite common and it is only the ability to use RS that is rare. This means there is likely to be much more competition for these non-RS carbohydrate sources from non-butyrate producing organisms. Interestingly in an experiment feeding mice the drug acarbose (Baxter et al., 2019a), an inhibitor of the human amyloglucosidase and to a lesser extent pancreatic amylase (Akkarachiyasit et al., 2010), non-RS rich diets produced a significant increase in butyrate. In these acarbose treated mice, it is expected that more starch makes it to the colon, analogous to our non-RS fermentations. The observed increase in butyrate is contrary to our *in vitro* fermentation results with non-RS, however, there is currently only limited information of the effect acarbose has on microbial amylases, though it is known to inhibit those from certain organisms (Santilli et al., 2018).

### Relationships Between RS Degraders, Starch Types, and Butyrate Production

Looking at RS degrading organisms in this study, five of the fecal inocula had only *B. adolescentis*, one had only *R. bromii* and five had both as detected RS degraders (Figure 6). Note that this only ∼50% prevalence for *R. bromii* would appear to be something of a sampling anomaly as the *Ruminococcus* genus of which *R. bromii* is the most prominent member in the human gut is generally considered to be a part of the core human gut microbiome with a high prevalence (Zhang et al., 2015; Falony et al., 2016). Nevertheless, it provides an interesting opportunity to explore the relative importance of the two main RS degrading taxa in butyrate production in the human gut. The HAM2 and HAM4 starches were particularly selective for *R. bromii* in these experiments, though its relatively low prevalence kept it from being detected as a differentially abundant OTU. The potato derived starches and non-resistant starches were the most selective for *B. adolescentis*. Human intervention trials with healthy adults have consistently found increases in *R. bromii* when people are fed HAM2 (Martínez et al., 2010; Maier et al., 2017; Vital et al., 2018; Baxter et al., 2019b) and increases in *B. adolescentis* when people are fed PS (Venkataraman et al., 2016; Alfa et al., 2018; Baxter et al., 2019b). For the other RS used in this study there is insufficient human trial data to draw strong conclusions, however, a recent trial using HAM4 and Tap found no significant increases in either RS degrader, though a closely related *Ruminococcus* OTU was significantly increased on HAM4 (Deehan et al., 2020). Given these trends it would appear that our *in vitro* system has well captured this competition between RS degraders on these various substrates.

It is intriguing that the only fecal inoculum that lacks *B. adolescentis* (T09) is also the one that had the most robust increases in butyrate production across treatments (Figure 2), even among the PS treatments where *R. bromii* overall did worse than *B. adolescentis*. A recent human intervention study with HAM2 and PS interventions similarly found that those individuals that had *R. bromii* increases were the most likely to experience butyrate increases (Baxter et al., 2019b). Indeed, while we did not find a strong correlation between *R. bromii* and butyrate production directly, there were numerous strong correlations between *R. bromii* and butyrate producers, particularly *F. prausnitzii* and *E. rectale* (Figure 7). However, the inoculum with the second highest ratio of *R. bromii* to *B. adolescentis* (T08) did not fare as well, producing increases in butyrate for only Bn and HAM2. Interestingly, it did have the largest increase in succinate of any of the inocula, primarily in the HAM4, RS3_Wh and Ap conditions (Supplementary Figure 4). This make the point that a high abundance of *R. bromii* is not in itself a guarantee of increased butyrate production. Indeed, the PS_Rb condition where *R. bromii* was supplemented in was one of the lowest butyrate producing treatments. Similarly, there were no universal trends among the inocula lacking *R. bromii* and those with both degraders, with mixed responses in each.

It should be noted that most butyrate producing bacteria do not exclusively produce butyrate and typically have multiple other potential fermentation products, particularly lactate. Additionally, some butyrate producers such as *Anaerostipes hadrus* and *Anaerobutyricum hallii* produce butyrate primarily through the consumption of lactate (Duncan et al., 2004). Thereby lactate levels are typically kept quite low in the colon and *B. adolescentis* can favor butyrate production through its production of lactate. However, lactate accumulation can be favored by a drop in pH (Belenguer et al., 2007; Wang et al., 2020), perhaps both by disfavoring lactate to butyrate converters and shifting butyrate producers over to lactate production. Indeed, butyrate production was both positively correlated with pH and negatively correlated with lactate (Figure 1). The end point pH reached in most fermentations in this study were at the low end of what is typical for the colon (Koziolek et al., 2015; Cremer et al., 2017) and stronger buffering toward neutral pH may have resulted in higher butyrate levels thereby forging a stronger link between RS degraders and butyrate production. This suggests that a three-way interplay between RS degraders, butyrate producers and pH is critical for driving butyrate production during RS fermentation. This also suggests that *R. bromii* based fermentations will be more likely to result in butyrate production due to the higher number of strong links between this organism and butyrate producers, though still dependent on pH.

### Interindividual Differences in Butyrate Production and Conclusion

In this study, we have examined the role of microbiome differences in driving interindividual differences in butyrate responses to resistant starch supplementation. Differences in diet composition or butyrate absorption were not examined in this *in vitro* system, however, while these may play a role *in vivo*, it is clear from these fermentations that there are other factors that are important in determining fecal butyrate levels. The levels of RS degrading organisms do not appear to have been a limiting factor in these fermentations as they were detected in all individuals at levels ranging from 1 to 15% of the total community by the end of the fermentations (Figure 6C). Furthermore, addition of RS degraders to PS fermentations for the two individuals with the lowest RS degrader content T02 and T10, only produced a significant increase in butyrate for T02 in the PS_Rb condition. Butyrate producers in total also don’t seem to have been limiting overall, making up at least 5% on average for all individuals and typically much higher. The makeup of the butyrate producers might be more important as butyrate producers in the Clostridiaceae and Peptostreptococcaceae were generally negatively associated with butyrate, while Lachnospiraceae and Ruminococcaceae producers were mostly positively associated with butyrate (Figure 7). The relative lack of lactate to butyrate producers may have also played a role (none were among the top 10 butyrate producers) and though none had significant positive correlations with butyrate it stands to reason that these bacteria would play an important role when *B. adolescentis* is the primary RS degrader. Production of different end products other than butyrate either by the butyrate producers themselves or by competing organisms seems to have played an important role in limiting butyrate production. This seems to have been almost entirely driven by lactate production as it was strongly negatively correlated with butyrate (Figure 1). Although we were not able to measure propionate accurately in this study, it does not seem likely that it was a dominant alternative to butyrate as there was not a notable number of propionate producers that increased significantly in any of the conditions and the Bacteroidetes, the main propionate producing phylum was generally found to decrease relative to the non-starch containing control (Figure 5). While it is not completely clear what is driving interindividual differences, it should be noted that all individual microbiomes had at least one RS that increased butyrate production and most multiple, though only T03 and T09 had increased butyrate for all RS sources. This suggests that individuals may be able to benefit by tailoring the RS source in their diet to their microbiome, though further studies will be required to link these *in vitro* results to butyrate production in humans and to identify key drivers for each RS.

## Data Availability Statement

The datasets presented in this study can be found in online repositories. The names of the repository/repositories and accession number(s) can be found below: https://www.ncbi.nlm.nih.gov/, PRJNA682640.

## Ethics Statement

The studies involving human participants were reviewed and approved by The Pennsylvania State University Institutional Review Board. The patients/participants provided their written informed consent to participate in this study.

## Author Contributions

JT conducted the experiments and performed part of the data analysis and writing. DC conceived of the experiments and performed part of the data analysis and writing. Both authors contributed to the article and approved the submitted version.

## Conflict of Interest

The authors declare that the research was conducted in the absence of any commercial or financial relationships that could be construed as a potential conflict of interest.

## Footnotes

1 https://mothur.org/wiki/miseq_sop/

2 https://github.com/microbiome/microbiome

3 https://cran.r-project.org/web/packages/vegan/index.html

4 https://github.com/leffj/mctoolsr

5 https://huttenhower.sph.harvard.edu/galaxy/

6 https://github.com/FrederickHuangLin/ANCOM

7 https://github.com/kassambara/ggpubr

8 https://github.com/umerijaz/microbiomeSeq

## Abbreviations

Ap: potato amylopectin
Bn: green banana flour
CS: corn starch
HAM2: high amylose maize starch (Hi-Maize^®^ 260 resistant starch)
HAM4: chemically cross-linked high amylose maize starch (VERSAFIBE^TM^ 2470 resistant starch)
PS: potato starch
PS_Ba: potato starch supplemented with *Bifidobacterium adolescentis*
PS_Rb: potato starch supplemented with *Ruminococcus bromii*
RS: resistant starch
RS3_Ex: retrograded potato starch
RS3_Wh: retrograded whole potatoes
SCFA: short chain fatty acids
Tap: chemically cross-linked tapioca starch (ActiStar^®^ RT)
Tn: tiger nut flour.
