# Supplementary figures and images for: In vitro Fermentation Reveals Changes in Butyrate Production Dependent on Resistant Starch Source and Microbiome Composition
Source: Front Microbiol. 2021 Apr 29;12:640253. doi: 10.3389/fmicb.2021.640253 (PMC8117019; doi:10.3389/fmicb.2021.640253)

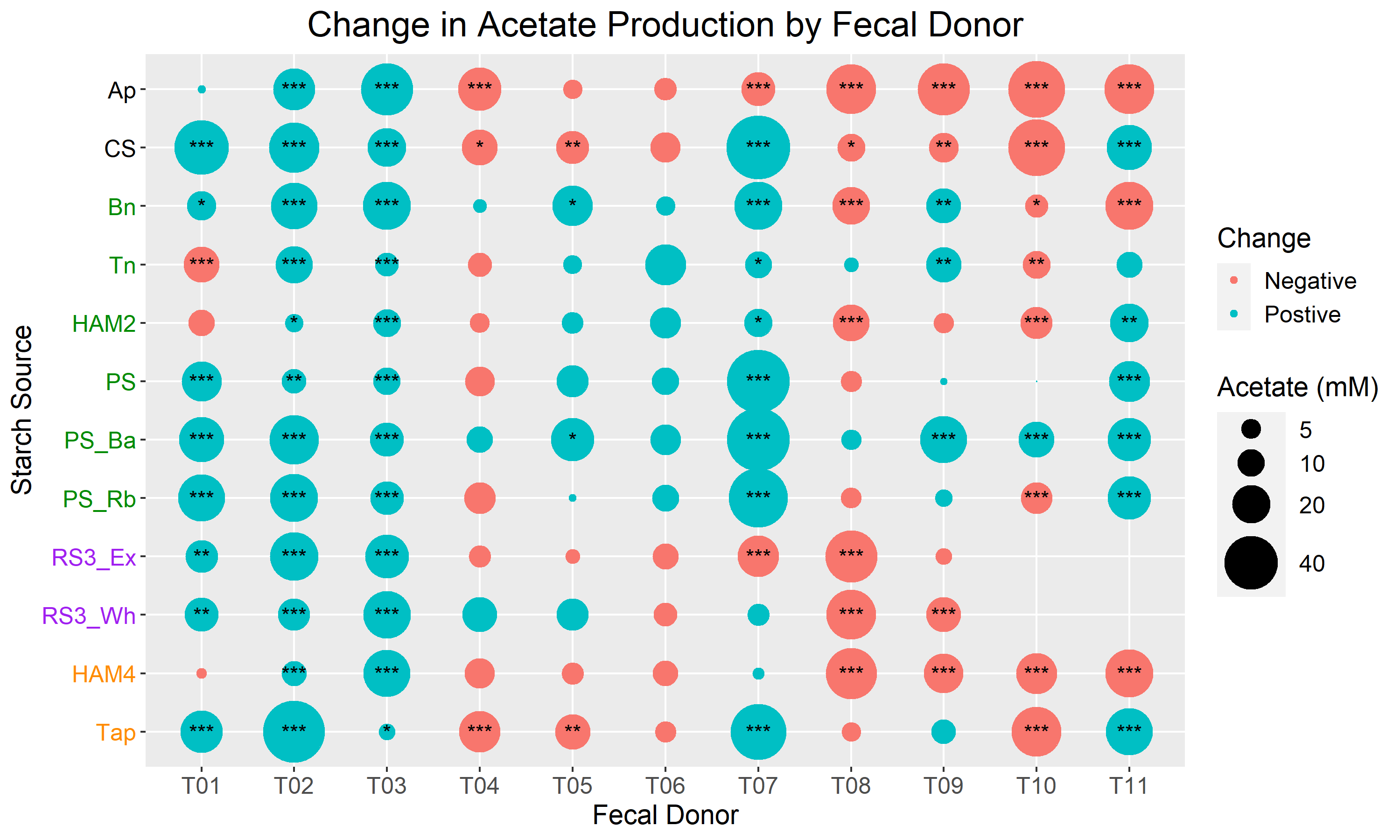

Supplement: Supplementary file 1 [file Image_1.TIFF]

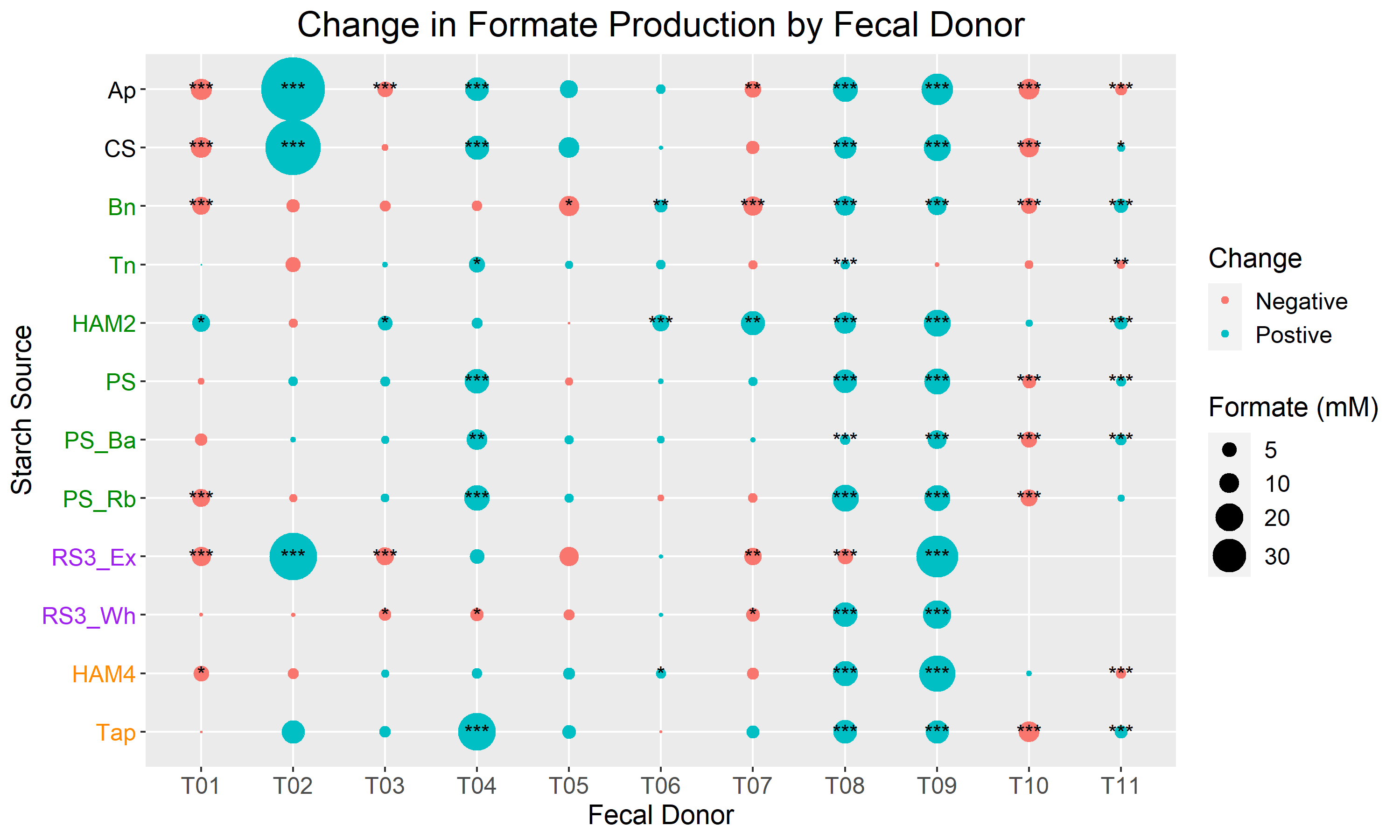

Supplement: Supplementary file 2 [file Image_2.TIFF]

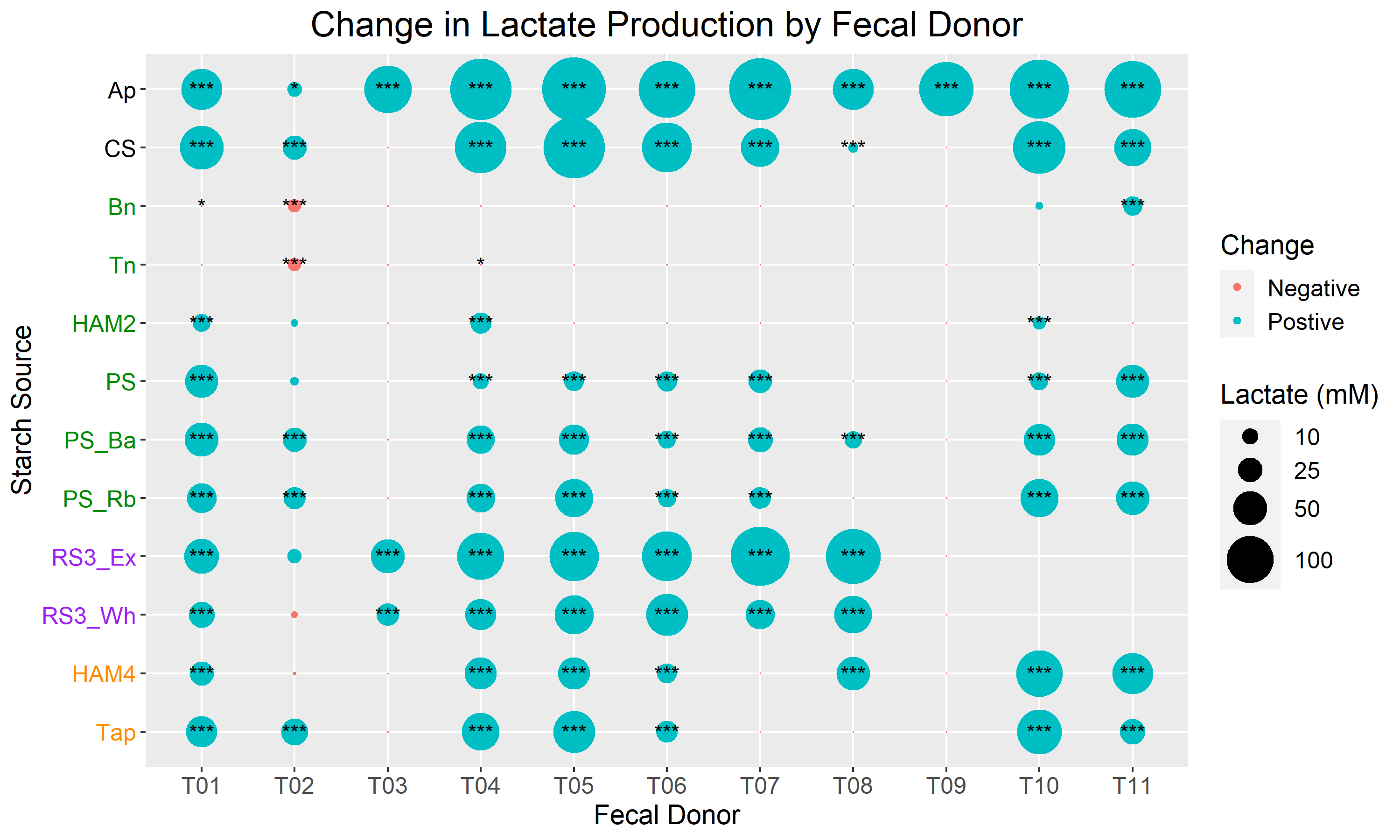

Supplement: Supplementary file 3 [file Image_3.TIFF]

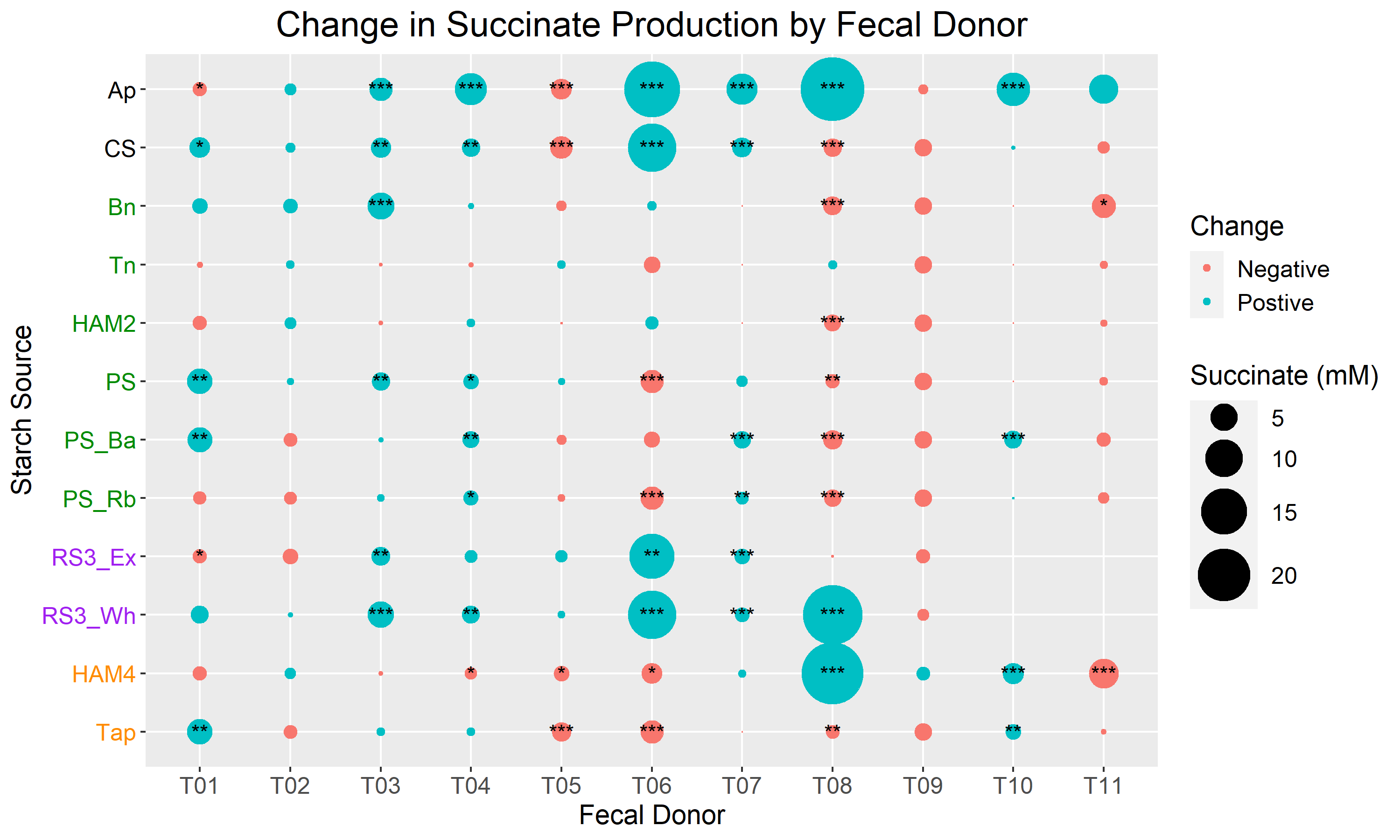

Supplement: Supplementary file 4 [file Image_4.TIFF]
