# Supplementary material for: In vitro Fermentation Reveals Changes in Butyrate Production Dependent on Resistant Starch Source and Microbiome Composition
Source: Front Microbiol. 2021 Apr 29;12:640253. doi: 10.3389/fmicb.2021.640253 (PMC8117019; doi:10.3389/fmicb.2021.640253)
Supplement: Supplementary file 5 [file Table_1.DOCX]

Supplementary Material

# Supplementary Tables

| Table S1\| Starches used in this study | | |
| --- | --- | --- |
| **Starch** | **Description** | **Resistant Starch Type** |
| Amylopectin (Ap) | Potato amylopectin | Non-resistant |
| Banana (Bn) | Green banana flour | Type 2 |
| Corn starch (CS) | Corn starch | Non-resistant |
| Hi amylose maize starch (HAM2) | Hi-Maize® 260 resistant starch (Ingredion) | Type 2 |
| Chemically cross-linked high amylose maize starch (HAM4) | VERSAFIBE^TM^ 2470 resistant starch (Ingredion) | Type 4 |
| Potato starch (PS) | Commercial potato starch | Type 2 |
| Retrograded potato starch (RS3) | Used as either starch that was first extracted from potatoes and then subjected to retrogradation (RS3_Ex) or whole potatoes that were subjected to retrogradation (RS3_Wh) | Type 3 |
| Chemically cross-linked tapioca starch (Tap) | ActiStar® RT (Cargill) | Type 4 |
| Tiger nut flour (Tn) | Commercial tiger nut flour | Type 2 |

| Table S2\| Identified butyrate producing organisms within the first 200 OTUs | |
| --- | --- |
| **OTU** | **Designation** |
| Otu00001 | Clostridium_perfringens |
| Otu00006 | Faecalibacterium_prausnitzii |
| Otu00027 | Roseburia_hominis |
| Otu00028 | X.Eubacterium._rectale |
| Otu00030 | Faecalibacterium_prausnitzii |
| Otu00031 | Clostridioides_difficile |
| Otu00033 | Flavonifractor_plautii |
| Otu00043 | Blautia_faecis |
| Otu00045 | Roseburia_inulinivorans |
| Otu00049 | Roseburia_faecis |
| Otu00054 | Faecalibacterium_prausnitzii |
| Otu00057 | Butyricicoccus_faecihominis |
| Otu00060 | Coprococcus_catus |
| Otu00063 | Clostridium_aciditolerans |
| Otu00071 | Faecalibacterium_prausnitzii |
| Otu00074 | Subdoligranulum_variabile |
| Otu00086 | Clostridioides_difficile |
| Otu00089 | Anaerostipes_hadrus |
| Otu00096 | Anaerobutyricum_hallii |
| Otu00115 | Coprococcus_eutactus |
| Otu00116 | Flintibacter_butyricus |
| Otu00122 | Clostridium_perfringens |
| Otu00126 | Clostridium_perfringens |
| Otu00138 | Intestinimonas_butyriciproducens |
| Otu00180 | Faecalibacterium_prausnitzii |
|  |  |
|  |  |

| Table S3\| Differences in fermentation parameters between treatments and control | | | | | | | | | | |
| --- | --- | --- | --- | --- | --- | --- | --- | --- | --- | --- |
| Starch^a^ | Acetate | | | Formate | | | Succinate | | |  |
|  | Value | Change^b^ | P | Value | Change^b^ | P | Value | Change^b^ | P |  |
| Ap | 21.41 | -13.05 | **0.010** | 23.23 | 12.9 | 0.561 | 9.31 | 7.18 | **0.039** |  |
| Bn | 42.95 | 8.49 | 0.934 | 8.85 | -1.48 | 0.721 | 2.1 | -0.03 | 0.991 |  |
| CS | 40.93 | 6.47 | 0.934 | 21 | 10.67 | 0.537 | 3.9 | 1.77 | 0.990 |  |
| HAM2 | 35.62 | 1.16 | 0.934 | 16.84 | 6.51 | 0.253 | 1.85 | -0.28 | 0.990 |  |
| HAM4 | 25.31 | -9.15 | 0.540 | 14.55 | 4.22 | 0.537 | 3.98 | 1.85 | 0.991 |  |
| PS | 45.95 | 11.49 | 0.788 | 14.73 | 4.40 | 0.489 | 2.31 | -0.08 | 0.991 |  |
| PS_Ba | 58.27 | 23.81 | 0.406 | 12.25 | 1.92 | 0.561 | 2.25 | 0.12 | 0.991 |  |
| PS_Rb | 46.7 | 12.24 | 0.906 | 13.8 | 3.47 | 0.561 | 1.41 | -0.72 | 0.990 |  |
| RS3_Ex | 32.06 | -2.4 | 0.906 | 18.23 | 7.9 | 0.721 | 3.72 | 1.59 | 0.990 |  |
| RS3_Wh | 37.52 | 3.06 | 0.934 | 12.45 | 2.12 | 0.85 | 7.71 | 5.58 | 0.070 |  |
| Tap | 41.71 | 7.25 | 0.934 | 17.92 | 7.59 | 0.43 | 1.79 | -0.34 | 0.990 |  |
| TN | 39.58 | 5.12 | 0.934 | 10.44 | 0.11 | 0.85 | 1.85 | -0.28 | 0.991 |  |
|  |  |  |  |  |  |  |  |  |  |  |
| Water | 34.46 | 0 |  | 10.33 | 0 |  | 2.13 | 0 |  |  |

^a^ Abbreviations are Ap – potato amylopectin, Bn – green banana flour, CS – non-resistant corn starch, HAM2 – high amylose maize starch, HAM4 – chemically cross-linked high amylose maize starch, PS – potato starch, PS_Ba – potato starch with fecal samples supplemented with *Bifidobacterium adolescentis*, PS_Rb – potato starch with fecal samples supplemented with *Ruminococcus bromii*, RS3_Ex – retrograded extracted potato starch, RS3_Wh – retrograded whole potato, Tap – chemically cross-linked tapioca starch, TN – tiger nut flour, Water – no starch control

^b^ change relative to the no-starch control (Water)

# Supplementary Figures

**Supplementary Figure 1.** Change in acetate production between control and treatments by fecal donor. In each case acetate production during the control fermentation for a given inoculum is subtracted from the acetate production in a given treatment with that same inoculum. Bubble size is proportional to the total change in acetate production, colored red for a decrease and blue for an increase. Y-axis labels of starch sources are colored by RS designation. Black is non-RS, green is RS2, purple is RS3, orange is RS4. Statistical significance of the difference is calculated by one-way ANOVA of the ln(x+1) transformed acetate values and FDR correction of P-values. Abbreviations are: Ap – amylopectin, CS – corn starch, Bn – green banana flour, Tn – tiger nut flour, HAM2 – high amylose maize starch, PS – potato starch, PS_Ba – potato starch with fecal samples supplemented with *Bifidobacterium adolescentis*, PS_Rb – potato starch with fecal samples supplemented with *Ruminococcus bromii*, RS3_Ex – retrograded extracted potato starch, RS3_Wh – retrograded whole potatoes, HAM4 – chemically cross-linked high amylose maize starch, Tap – chemically cross-linked tapioca starch.

**Supplementary Figure 2.** Change in formate production between control and treatments by fecal donor. In each case formate production during the control fermentation for a given inoculum is subtracted from the formate production in a given treatment with that same inoculum. Bubble size is proportional to the total change in formate production, colored red for a decrease and blue for an increase. Y-axis labels of starch sources are colored by RS designation. Black is non-RS, green is RS2, purple is RS3, orange is RS4. Statistical significance of the difference is calculated by one-way ANOVA of the ln(x+1) transformed formate values and FDR correction of P-values. Abbreviations are: Ap – amylopectin, CS – corn starch, Bn – green banana flour, Tn – tiger nut flour, HAM2 – high amylose maize starch, PS – potato starch, PS_Ba – potato starch with fecal samples supplemented with *Bifidobacterium adolescentis*, PS_Rb – potato starch with fecal samples supplemented with *Ruminococcus bromii*, RS3_Ex – retrograded extracted potato starch, RS3_Wh – retrograded whole potatoes, HAM4 – chemically cross-linked high amylose maize starch, Tap – chemically cross-linked tapioca starch.

**Supplementary Figure 3.** Change in lactate production between control and treatments by fecal donor. In each case lactate production during the control fermentation for a given inoculum is subtracted from the lactate production in a given treatment with that same inoculum. Bubble size is proportional to the total change in lactate production, colored red for a decrease and blue for an increase. Y-axis labels of starch sources are colored by RS designation. Black is non-RS, green is RS2, purple is RS3, orange is RS4. Statistical significance of the difference is calculated by one-way ANOVA of the ln(x+1) transformed lactate values and FDR correction of P-values. Abbreviations are: Ap – amylopectin, CS – corn starch, Bn – green banana flour, Tn – tiger nut flour, HAM2 – high amylose maize starch, PS – potato starch, PS_Ba – potato starch with fecal samples supplemented with *Bifidobacterium adolescentis*, PS_Rb – potato starch with fecal samples supplemented with *Ruminococcus bromii*, RS3_Ex – retrograded extracted potato starch, RS3_Wh – retrograded whole potatoes, HAM4 – chemically cross-linked high amylose maize starch, Tap – chemically cross-linked tapioca starch.

**Supplementary Figure 4.** Change in succinate production between control and treatments by fecal donor. In each case succinate production during the control fermentation for a given inoculum is subtracted from the succinate production in a given treatment with that same inoculum. Bubble size is proportional to the total change in succinate production, colored red for a decrease and blue for an increase. Y-axis labels of starch sources are colored by RS designation. Black is non-RS, green is RS2, purple is RS3, orange is RS4. Statistical significance of the difference is calculated by one-way ANOVA of the ln(x+1) transformed succinate values and FDR correction of P-values. Abbreviations are: Ap – amylopectin, CS – corn starch, Bn – green banana flour, Tn – tiger nut flour, HAM2 – high amylose maize starch, PS – potato starch, PS_Ba – potato starch with fecal samples supplemented with *Bifidobacterium adolescentis*, PS_Rb – potato starch with fecal samples supplemented with *Ruminococcus bromii*, RS3_Ex – retrograded extracted potato starch, RS3_Wh – retrograded whole potatoes, HAM4 – chemically cross-linked high amylose maize starch, Tap – chemically cross-linked tapioca starch.
